# Post-cardiac injury syndrome occurred two months after permanent dual-chamber pacemaker implantation

**DOI:** 10.1186/s12872-023-03252-5

**Published:** 2023-05-19

**Authors:** Ruiqi Zhang, Jiali Du, Meilin Liu

**Affiliations:** Department of Geriatrics, Peking University First Hospital, 100034 Beijing, People’s Republic of China

**Keywords:** Post-cardiac injury syndrome, Pacemaker implantation, Complications, Cardiac tamponade, Pericardial effusion

## Abstract

**Background:**

Post cardiac injury syndrome (PCIS) is characterized by the development of pericarditis with or without pericardial effusion due to a recent cardiac injury. The relatively low incidence makes diagnosis of PCIS after implantation of a pacemaker easily be overlooked or underestimated. This report describes one typical case of PCIS.

**Case presentation:**

We present a case report of a 94-year-old male with a history of sick sinus syndrome managed with a dual-chamber pacemaker who presented with PCIS after two months of pacemaker implantation. He gradually developed chest discomfort, weakness, tachycardia and paroxysmal nocturnal dyspnea and cardiac tamponade after two months of pacemaker. Post-cardiac injury syndrome related to dual-chamber pacemaker implantation was considered based on exclusion of other possible causes of pericarditis. His therapy was drainage of pericardial fluid and managed with a combination of colchicine and support therapy. He was placed on long-term colchicine therapy to prevent any recurrences.

**Conclusion:**

This case illustrated that PCIS can occur after minor myocardial injury, and that the possibility of PCIS should be considered if there is a history of possible cardiac insult.

## Introduction

Post cardiac injury syndrome (PCIS) is characterized by the development of pericarditis with or without pericardial effusion due to a recent cardiac injury. In addition to myocardial infarction, PCIS has been shown to be induced by pericardiotomy and blunt trauma, as well as by minor insults to the heart, such as coronary intervention, insertion of pacemaker leads, or radiofrequency ablation [1–6]. However, since PCIS induced by insertion of a pacemaker or by coronary intervention is relatively uncommon, it is possible to miss this as an important differential diagnosis. Herein, we present a case of a 94-year-old male patient with a history of sick sinus syndrome treated with permanent dual-chamber pacemaker implantation, who displayed multiple symptoms of post-cardiac injury syndrome.

## Case presentation

**Fig. 1:**
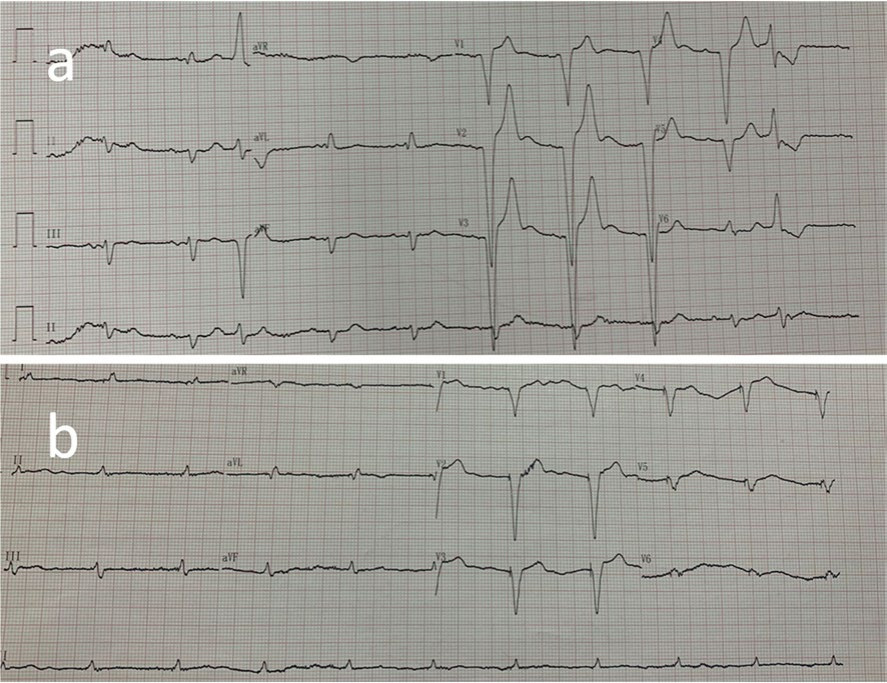
Electrocardiograph (**a**: one day after pacemaker implantation, b: this time, two months after pacemaker implantation) **b**: Pacemaker rhythm, no abnormalities compared to a

**Fig. 2:**
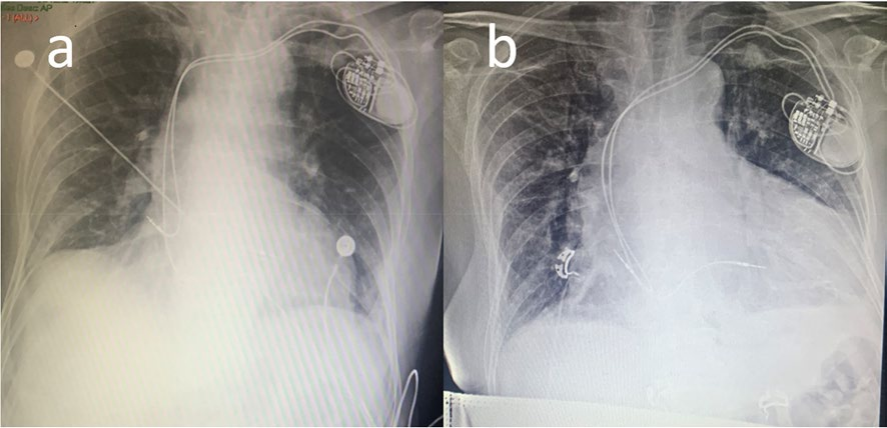
Chest X-ray (**a**: one day after pacemaker implantation, b: this time, two months after pacemaker implantation) **b**: Multiple patchy high-density shadows in both lungs, enlarged heart shadow, blurred left costophrenic angle

**Fig. 3:**
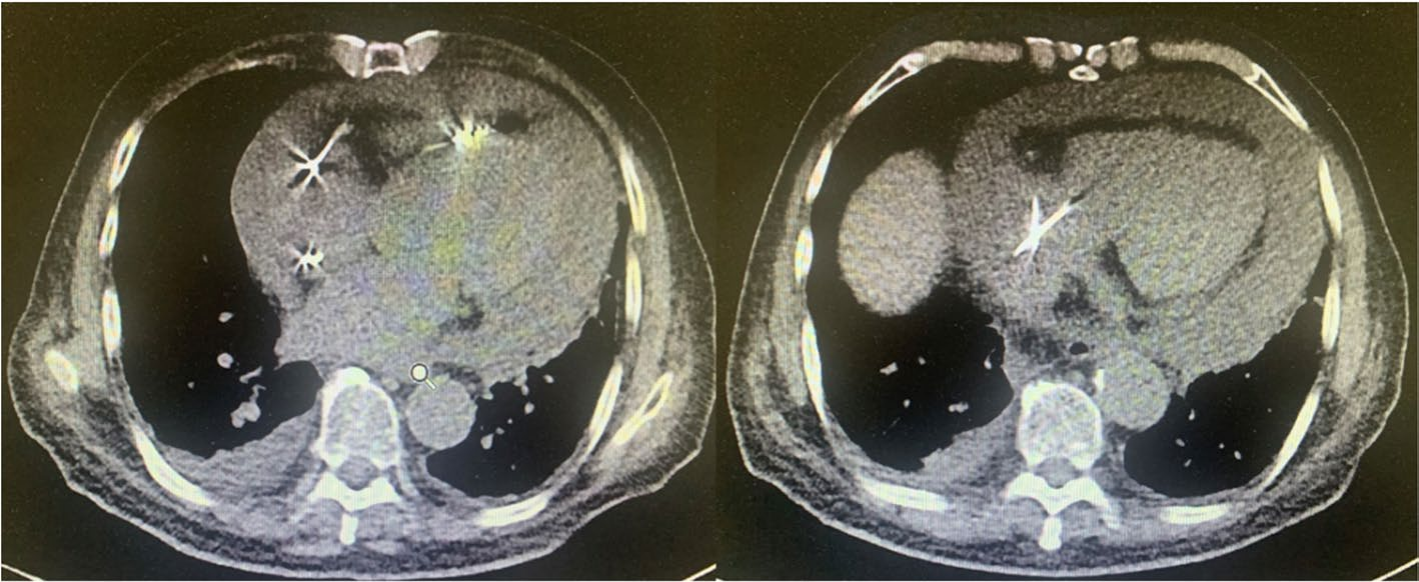
Chest CT: Large pericardium effusion, bilateral pleural effusion, partial expansion of both lungs, rule out perforation

**Fig. 4:**
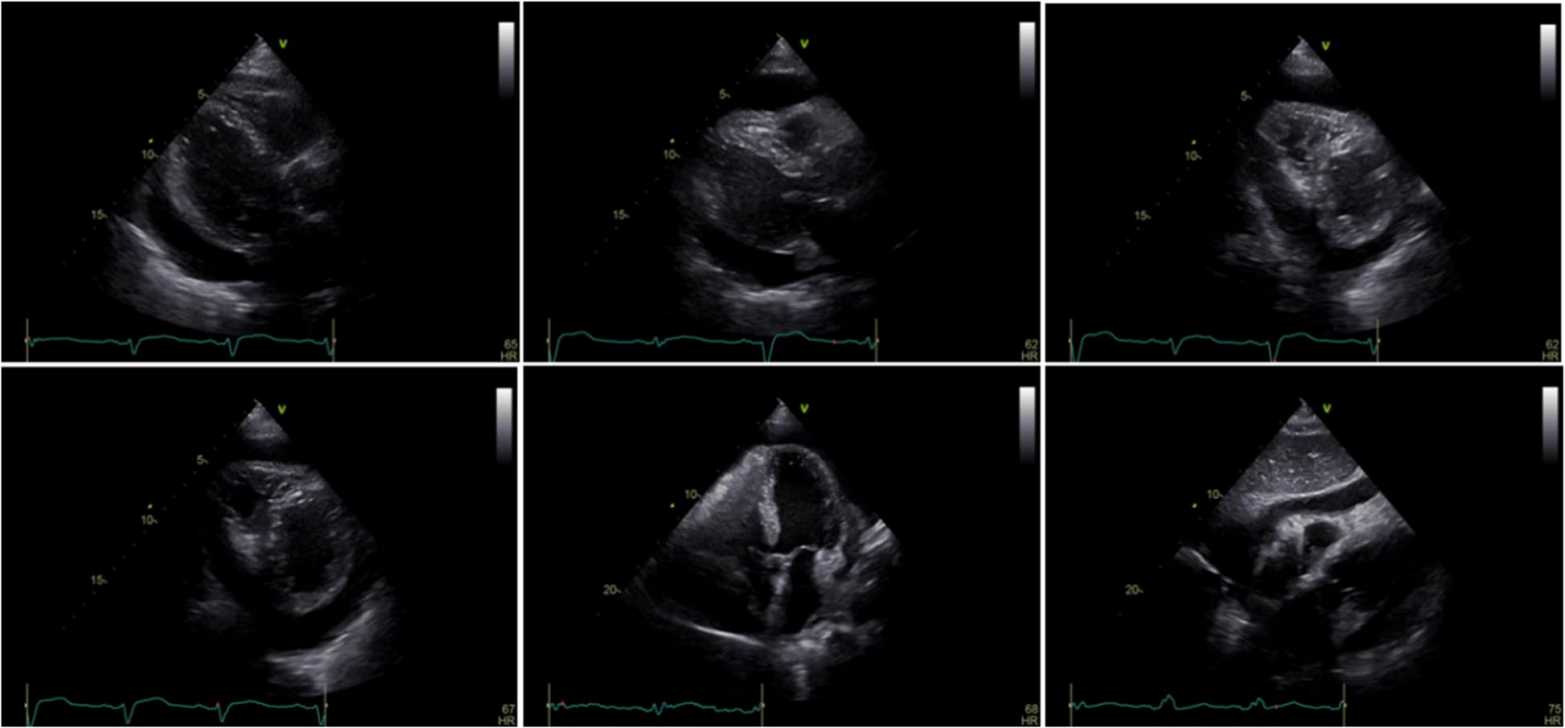
UCG: A large amount of pericardial effusion, diastolic collapse of right ventricle and right atrium, heart showed a swing sign, the inferior vena cava diameter widened (2.3 cm) and the difference of inspiratory retraction, left atrial enlargement (4.09 cm *5.54 cm *4.42 cm), EF 59%, PASP 48 mmHg

**Fig. 5:**
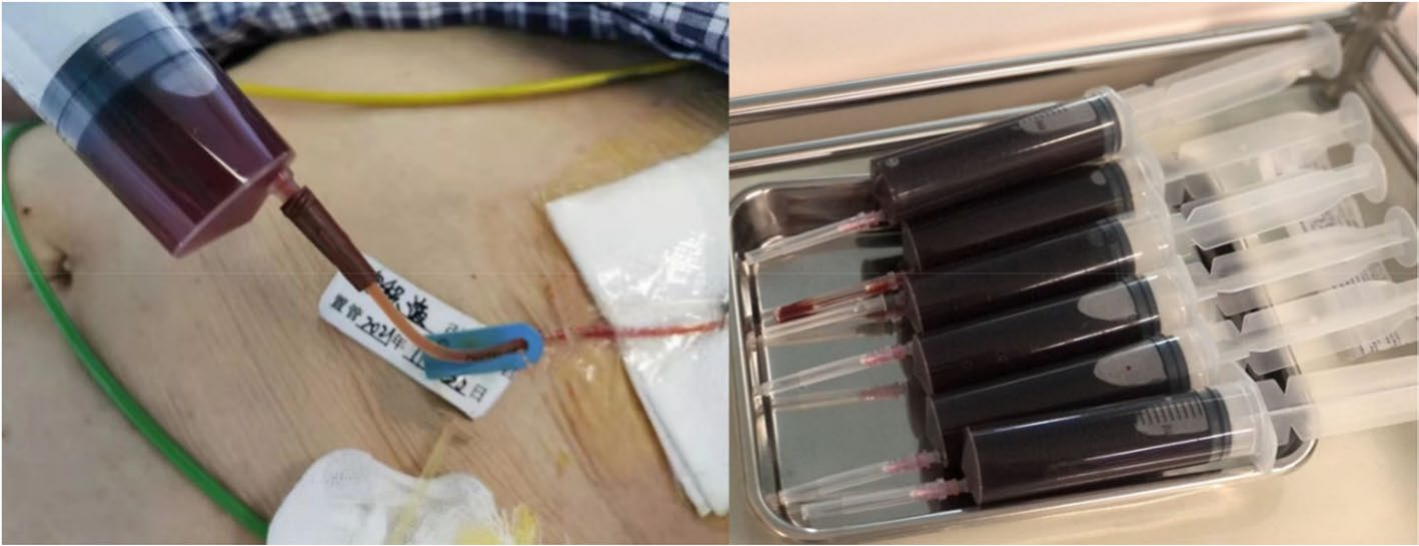
Pericardium puncture and drainage (first 610 ml and second 95 ml)

A 94-year-old Chinese Han male presented to the clinic with chief complaints of dyspnea, chest pain, and generalized weakness, the vital signs were BP 140/77 mmHg and HR 60 bpm on November 29, 2021. Previous history includes hypertension which treated by Hydrochlorothiazide, and prostate cancer which treated by Bicalutamide and Goserelin acetate. Surgical history includes sclerotherapy for varicose veins in the left leg ten years ago, and a permanent dual-chamber pacemaker (Medtronic A3DR01, the leads was active fixation and the ventricular catheter location was septal) that was implanted two months ago for sick sinus syndrome. Further examination is as follows: Arterial blood gas: PH 7.44, PCO_2_ 28 mmHg, PO_2_ 73 mmHg, K 4.4mmo1/L, Lac 0.6 mmol/L, HCO_3_- 19.0 mmol/L. Blood routine tests: WBC 6.52 × 10^9^/L, Hb 111 g/L, PLT 230 × 10^9^/L, NE% 77.9%. Liver and kidney function: ALT 60 IU/L, AST 71 IU/L, ALB 34.7 g/L, Scr 140.20umo1/L, eGFR 36.726 ml/min/1.73 m^2^ .Cardiac enzyme: CK-MB 3.5 ng/ml, hsTnI 140 ng/L, BNP 350 pg/ml. Coagulation: PT 13.4 s, APTT 34.8 s, FIB-C 4.87 g/L, D-D 3.03 mg/L. CRP 121 mg/L. PCT 0.11 ng/ml. The workup for infectious or autoimmune etiology, including TB-spot, HIV, herpes simplex virus (HSV), rapid plasma regains (RPR), echovirus, cytomegalovirus (CMV), fungal culture, antinuclear antibodies (ANA), and rheumatoid factor was negative. The pacemaker showed normal function. Electrocardiograph (ECG): Pacemaker rhythm, no abnormalities compared to his ECG after pacemaker (Fig. 1). Chest X-ray: Multiple patchy high-density shadows in both lungs, enlarged heart shadow, blurred left costophrenic angle (Fig. 2). Chest CT: Large pericardium effusion, bilateral pleural effusion, partial expansion of both lungs, rule out perforation (Fig. 3). Echocardiogram: A large amount of pericardial effusion, diastolic collapse of right ventricle and right atrium, heart showed a swing sign, the inferior vena cava diameter widened (2.3 cm) and the difference of inspiratory retraction, left atrial enlargement (4.09 cm *5.54 cm *4.42 cm), EF 59%, PASP 48 mmHg (Fig. 4). Then he underwent low flow oxygen, intravenous Ceftriaxone (Rocephin), diuretic treatment, and pericardium puncture. His pericardium effusion was drainage 705 ml totally (Fig. 5, first 610 ml and second 95 ml). His pericardial histologic studies showed chronic inflammation with reactive changes, and they were negative for acid-fast bacilli and malignant cells. Cytology of pericardial fluid revealed hemorrhagic fluid (Hb 18 g/L) with nucleated cells (2756/mm^3^). Given his clinical manifestations on a background of unremarkable past medical history, and pacemaker implantation, circumstantial evidence raised suspicion for post cardiac injury syndrome. Tumor, tuberculosis or pacemaker lead perforation were excluded based on the above findings. As a result, he was started on a trial of colchicine 0.25 mg once a day, which he tolerated well without further recurrences of his symptoms. His follow up echocardiogram showed decreasing gradually pericardial effusion one month after discharge (Table 1).

**Table 1:** Changes of pericardial effusion of echocardiography during treatment and follow-up

Date	Posterior pericardium (cm)	Anterior pericardium (cm)	Lateral pericardium (cm)	Right roof (cm)
11.29.2021	2.19	2.41	2.53	1.58
11.30.2021	First pericardium puncture and drainage 610 ml			
12.01.2021	1.70	0.50	1.80	0.64
12.02.2021	Second pericardium puncture and drainage 95 ml			
12.07.2021	0.39	0.33	0.49	0.30
01.06.2022	1.04	0.54	0.40	0.70
01.17.2022	0.61	0.36	0.51	0.55
02.20.2022	0.19	-	0.27	0.4

## Discussion

Post cardiac injury syndrome (PCIS) is characterized by the development of pericarditis with or without pericardial effusion due to a recent cardiac injury. The pathogenesis of PCIS is unclear, it may be an autoimmune process after heart injury, with antigens derived from damaged myocardial tissue.

Causes of PCIS include myocardial infarction, pericardiotomy, blunt trauma, and minor damage to the heart, such as coronary intervention, insertion of pacemaker leads, or radiofrequency ablation [1, 2]. Post-pacemaker insertion pericarditis is a rare type of PCIS that occurs in 1% to 2% of patients after pacemaker implantation [7]. Previous reports have shown that PCIS can occur within hours or days, or as early as 5–56 days after the procedure [8]. A patient’s medical history is essential for the recognize and diagnosis of PCIS, and patients undergoing the procedure should be followed up regularly from 1 to 3 months after the procedure [9]. The patient in our case is a 94-year-old male with a history of sick sinus syndrome managed with a dual-chamber pacemaker who presented with PCIS after two months of pacemaker implantation.

The mechanism of PCIS in general and post pacemaker insertion pericarditis in particular is still not well understood. Advanced age, female gender and the use of active fixation leads, a temporary transvenous pacemaker or steroid use are independent risk factors for the development of post pacemaker insertion pericarditis [10]. A proposed theory is that injury to mesothelial pericardial cells induces an immune response, leading to immune complex deposition in the pericardium, pleura, and lungs, which causes an inflammatory response [1, 7, 11]. The auto-immune nature of PCIS is supported by clinical features such as the latent period between the insult and symptoms, elevation of inflammatory markers, good response to NSAIDs, and a tendency to recur [4]. However, unlike other autoimmune diseases, circulating anti-cardiac antibodies are not detected until 14 days after the onset of PCIS, rather than at the initial diagnosis, and thus are not helpful in the diagnosis of PCIS [12].

The clinical manifestations of PCIS are pleurisy chest pain, fever, pericardial effusion and/or pleurisy with or without pleural effusion, and elevated reactants in the acute phase. Although almost all patients with PCIS have pericardial effusion, not all patients with pericardial effusion have symptoms or require treatment [10]. Distant heart sounds can be heard on physical examination, with signs of pericardial or pleural friction and pleural effusion. Chest imaging and echocardiography confirmed the presence of pleural and/or pericardial effusion and lead location. The diagnosis of post-cardiac injury syndrome after dual pacemaker implantation can only be diagnosed when other common infectious, autoimmune, or malignant causes have been excluded. An important differential diagnosis of PCIS is overt or minor lead perforation, which is also a common complication of pacemaker implantation [13]. There is no clear standard to distinguish pacemaker lead perforated from PCIS without perforation. Capture threshold increases, R wave amplitude decreases, lead impedance increases or decreases significantly, indicating lead perforation [6]. However, normal pacemaker function does not rule out the possibility of a perforated lead. In rare cases, lead perforation may be visible on imaging [7]. The patient in our case has typical symptoms and laboratory results consistent with the characteristics of PCIS, and was stable after 3 months of treatment according to the treatment regimen of PCIS. Reexamination of the UCG showed no increase in pericardial effusion, so the diagnosis of PCIS was considered.

The treatment of PCIS consists of similar treatment to other cases of acute pericarditis. The first-line therapy includes a combination of non-steroidal anti-inflammatory drugs and colchicine [8, 9, 14, 15]. Patients who do not tolerate NSAIDs and colchicine therapy or have a resolution of symptoms may be given a course of corticosteroids, which are tapered over weeks as the symptoms resolve [1, 9, 10, 16, 17]. If the patient develops pericardial effusion leading to cardiac tamponade, treatment with a pericardial window or surgical drainage may be necessary. Our patient showed adequate improvement with colchicine treatment and pericardium puncture though we reduced his colchicine dosage because of his advanced age. PCIS may be an immune process produced after heart injury, and attention to bed rest, avoid fatigue and strengthen nutritional support after heart injury may reduce the risk of PCIS.

## Conclusions

Post pacemaker insertion pericarditis is a rare form of PCIS, and it usually presents within one month from pacemaker implantation with symptoms and signs of pericarditis. Diagnosis of PCIS is usually based on exclusion of other possible causes of pericarditis. Although PCIS responds well to NSAIDs and colchicine therapy, and has favorable prognosis, delayed diagnosis may result in potential serious complications such as cardiac tamponade. Therefore, its early detection is of clinical importance. Attention to bed rest after heart injury, avoid fatigue, strengthen nutritional support, may reduce the risk of incidence of PCIS.

## Data Availability

The datasets used and/or analyzed during the current study available from the corresponding author on reasonable request.
